# MicroRNAs and Their Roles in Breast Cancer Bone Metastasis

**DOI:** 10.1007/s11914-021-00677-9

**Published:** 2021-04-08

**Authors:** Margherita Puppo, Manoj K. Valluru, Philippe Clézardin

**Affiliations:** 1 Present Address: Oncology and Metabolism Department (OMD), Medical School, University of Sheffield, Sheffield, UK; 2 Infection Immunity and Cardiovascular Department (IICD), Medical School, University of Sheffield, Sheffield, UK; 3 INSERM, Research Unit UMR S1033, LyOS, Faculty of Medicine Lyon-Est, University of Lyon 1, Lyon, France

**Keywords:** MicroRNAs, Breast cancer, Bone metastasis, miRNA therapy, Biomarkers

## Abstract

Bone metastasis occurs in advanced stages of breast cancer, worsening the quality of life and increasing the mortality of patients. Current treatments for bone metastasis are only palliative, and efficient therapeutic targets need to be still identified. MicroRNAs (miRNAs) are a large class of small non-coding RNAs that regulate gene expression within cells. Interestingly, the expression of certain miRNAs has been associated with several stages of bone metastasis progression, highlighting the importance of these small RNAs during the course of the metastatic disease. In this review, we aim to summarise the most recent findings on miRNAs and their mRNA targets in driving breast cancer bone metastasis. Furthermore, we discuss the possibility to use miRNAs as direct therapeutic targets or as advanced therapies for breast cancer bone metastasis, as well as their potential as predictive biomarkers of bone metastasis for an early diagnosis and a better tailoring of therapies for cancer patients.

## Introduction

Bone metastasis is a frequent complication of breast cancer [1, 2]. Bone metastasis results from the spreading of primary cancer cells in the bone microenvironment [3]. In bone, cancer cells need to adapt to a new micro-environment, which is highly vascularised, and to interact with bone resident cells (osteoblasts, osteocytes and osteoclast) as well as other cells present in the bone marrow (*e.g*. immune system cells) [3]. The presence of a permissive soil called “pre-metastatic niche” in the bone micro-environment and osteomimicry—the process for which “foreigner” cells mimic bone resident cells—are key steps for cancer cells to successfully seed in bone [4, 5]. Interestingly, cancer cells homing in bone can stay dormant for a long time, usually by exiting and re-entering in a low-proliferative state, until they eventually receive a signal that triggers their reactivation [6]. Once cancer cells start to proliferate, they first form undetectable micro-metastases, which then evolve in clinically detectable bone metastases [7]. As a consequence of the presence of a macro-metastasis or multiple metastases in bone, cancer patients suffer of bone fractures, spinal cord compression, bone pain and disability due to a weaker bone structure [8]. About 30% of breast cancer patients with advanced disease develop metastases, with a high prevalence of metastases to the bone compared to other distant sites [9]. Breast cancer patients with bone metastasis usually present osteolytic lesions (bone destruction) in the vertebrae and weight-bearing bones, which may lead to pathological fractures that require surgical interventions [10]. Current treatments for bone metastasis involve systemic therapies, such as chemotherapy and endocrine treatments, to slow down the proliferation rate of cancer cells, bone targeted agents, such as bisphosphonates or Denosumab (a monoclonal antibody), to inhibit excessive cancer-associated bone destruction and the use of bone seeking radionucleotides [11]. However, although these treatments can improve the quality of life of cancer patients with bone metastasis, they are only palliative [11], and there is therefore a need for novel therapeutic or diagnostic interventions to prevent bone metastasis formation.

**Fig. 1:**
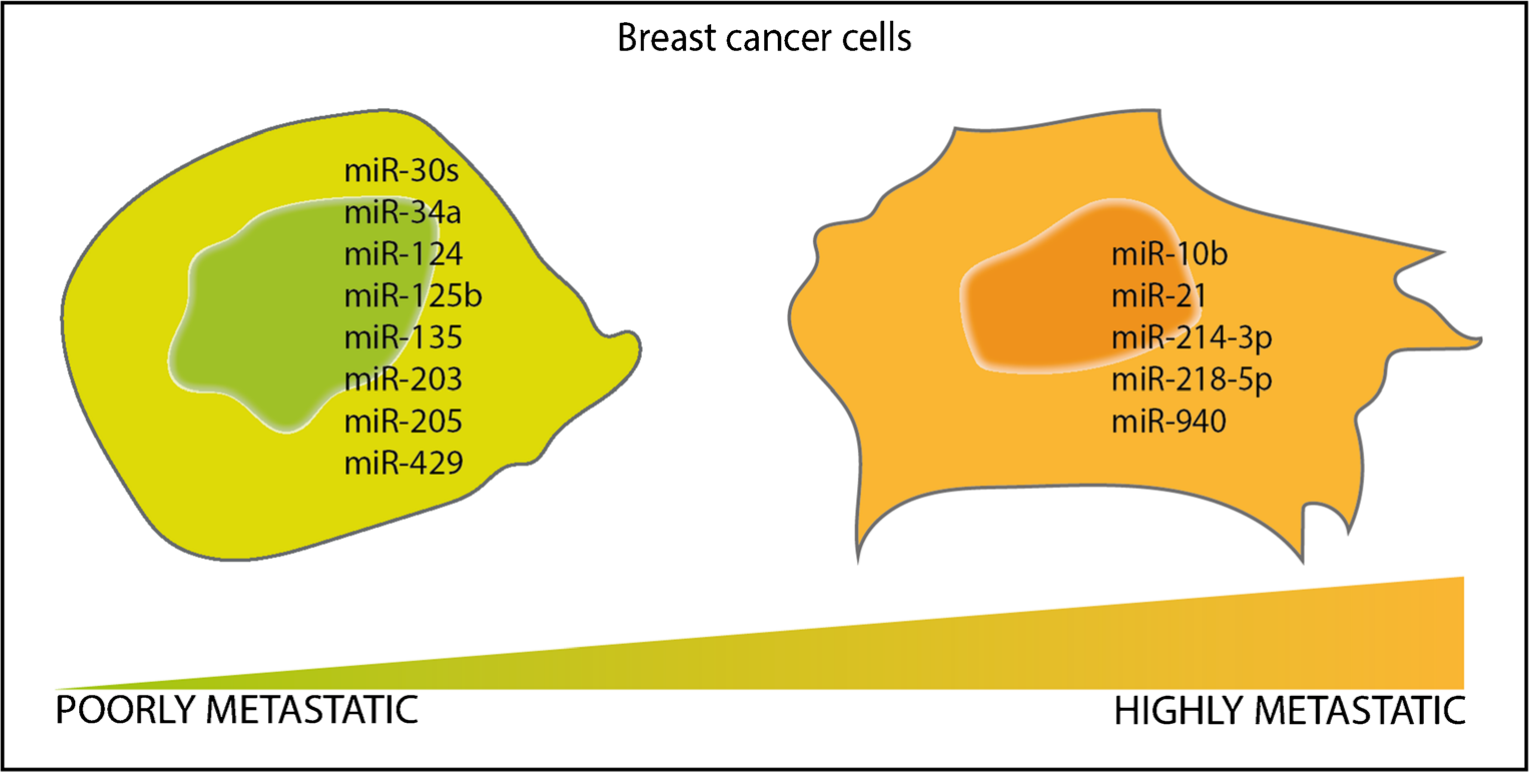
MiRNAs expression in breast cancer cells. Breast cancer cells with a different metastatic potential express different miRNAs that contribute to their phenotype. Here, we show a schematic representation of the expression of those miRNAs in poorly or highly metastatic breast cancer cells

MicroRNAs (miRNAs) are a large class of short non-coding RNAs that regulate gene expression within cells. The biogenesis of miRNAs is complex and requires the activity of different enzymes to obtain a mature form that complexes with Argonaute proteins (AGO) to form an effector complex called RNA-induced silencing complex (RISC). RISC recognises complementary sequences with the ‘guide’ miRNA sequence on mRNAs targets to mediate their degradation [12]. Of note, the post-transcriptional regulation of miRNAs is very complex within cells since one single miRNA can target hundreds of different mRNA targets. Moreover, the expression of a specific mRNA can be regulated by several different miRNAs [13]. MiRNA regulation activity in cells is therefore of crucial importance to maintain physiological functions, and their dysregulation has been observed in cancer cells [14]. By definition, oncomiRs are miRNAs which expression promotes tumorigenesis by inhibiting the translation of oncosuppressor genes, while oncosuppressor miRNAs have an opposite role inhibiting oncogene expression [15]. The expression of oncomiRs or oncosuppressor miRNAs is often time related, and they regulate and promote different steps of tumour progression from early to advanced stages. In bone metastasis, several miRNAs have been reported to act as drivers of the molecular changes within cells [16]. The expression of some miRNAs has been associated with an invasive and aggressive phenotype of cancer cells that promote their dissemination, or with their preference to metastasise in bone. Here, we will discuss the most recent findings over the last 5 years on miRNAs that regulate the formation and development of breast cancer bone metastasis (Table 1, Fig. 1), and how our current knowledge on miRNAs can be used for innovative therapies and/or development of diagnostic tools.

**Table 1:** MiRNAs controlling breast cancer bone metastasis formation

OncomiRs					
MiRNA name:	miR-10b	Validated targets:	*HoxD10*	References:	[17, 18]
	miR-21		*Pten, Pdcd4, Spry2*		[19]
	miR-214-3p		*Traf3*		[20–22]
	miR-218-5p		*Sost, Sfrp2*		[23, 24]
	miR-940				[25]
Oncosuppressor miRNAs					
MiRNA name:	miR-30s	Validated targets:	*Cdh11, Itga5*	References:	[26–29]
	miR-34a				[30, 31]
	miR-124		*Il-11*		[34]
	miR-125b				[35]
	miR-135		*Smad5*		[36]
	miR-203				[36]
	miR-205		*Itga5*		[37]
	miR-429		*Crkl*		[40]

## OncomiRs in Breast Cancer Bone Metastasis

### miR-10b

MiR-10b has been identified as an important oncomiR associated with the metastatic potential of breast cancer cells, promoting tumour cell migration and invasion to secondary organs, such as lung [17]. In early stages of breast cancer metastasis progression, miR-10b promoted by TWIST1 inhibits the expression of the transcription factor homeobox D10, thus leading to the over-expression of a pro-metastatic factor, RHOC, in cancer cells [17]. Recently, it has also been demonstrated that, in a bone-seeking sub-population of metastatic breast cancer cells, TWIST1 promotes bone metastasis formation [18]. Interestingly, the transfection of a miR-10b antagomiR in these TWIST1-expressing bone-seeking breast cancer cells inhibits the development of experimental bone metastasis in mice [18]. These findings should encourage the development of targeted therapies that specifically inhibit TWIST1 and/or miR-10b expression levels in cancer cells in order to reduce the risk of bone metastasis formation in breast cancer patients.

### miR-21

The oncomiR miR-21 is highly expressed in several cancers and associated with poor prognosis in patients with cancer. In breast cancer, miR-21 promotes metastatic breast cancer cells to colonise bone and form macro-metastasis [19]. Mechanistically, lysophosphatidic acid (LPA_1_), a bioactive lipid that promotes cancer progression and bone metastasis, stimulates miR-21 expression in breast cancer cells *via* a LPA_1_/Pi3K/ZEB1-dependent signalling pathway [19]. The inhibition of miR-21 expression results in a decreased capacity of breast cancer cells to migrate in vitro, and a decrease of bone metastasis formation in vivo [19]. A similar effect is obtained with the silencing of LPA_1_ or ZEB1 reinforcing the evidence that LPA_1_ and ZEB1 are upstream regulators of miR-21 [19]. Thus, by acting on multiple targets on this pathway is possible to prevent metastatic cells to migrate to bone.

### miR-214-3p

The osteoclast-derived miR-214-3p contributes to breast cancer bone metastasis [20]. Metastatic breast cancer cells usually induce osteolytic lesions by increasing osteoclast-mediated bone resorption, thereby causing excessive bone destruction [16]. The expression level of miR-214-3p in pathological bone fracture specimens from breast cancer patients with bone metastases is higher than that in breast cancer patients without osteolytic bone metastasis and even more elevated in comparison to miR-214-3p levels in bone fracture specimens from cancer-free patients [20]. In *miR-214-3p* knockout nude mice, the osteoclast activity is reduced, and the osteolytic bone metastasis development after breast cancer cell inoculation is prevented in comparison to normal nude mice bearing breast cancer bone metastases [20]. MiR-214-3p directly targets *Traf3*, an inhibitor of osteoclast differentiation, thereby promoting osteoclast-mediated bone resorption [20]. Interestingly, the inhibition of the osteoclastic miR-214-3p has a therapeutic effect in animal models by attenuating the extent of osteolytic bone metastases [20]. Of note, osteoclast-derived exosomal miR-214-3p can also transfer to osteoblasts to inhibit bone formation [21], and tumour-derived miR-214 promotes breast cancer cell dissemination in vivo [22]. Collectively, these studies [20–22] strongly suggest that inhibition of miR-214-3p may be a strategy for treating osteolytic lesions in metastatic breast cancer.

### miR-218-5p

MiR-218-5p is an oncomiR that favours the metastatic dissemination of triple-negative breast cancer (TNBC) cells in bone [23]. MiR-218-5p expression levels are increased in bone metastasis patients’ samples in comparison to healthy bone controls and primary breast tumours [23], suggesting its involvement in bone metastasis progression. In line with these findings, the metastatic MDA-MB-231 cell line shows increased levels of miR-218-5p in comparison to a non-malignant cell line (MCF10A) and a non-metastatic cell line (MCF7) [23]. The over-expression of miR-218-5p in MDA-MB-231 cells increases cell proliferation in vitro, whereas miR-218-5p downregulation results in a reduced proliferation of MDA-MB-231 cells in vitro and reduced tumour growth and osteoclast activity in vivo [23]. MiR-218-5p directly targets SOST and SFRP2, two inhibitors of the Wnt-signalling pathway, elucidating the mechanism by which this miRNA operates within cancer cells [23]. Also, low expression levels of miR-218-5p results in a downregulation of genes involved with bone metastasis and osteoclast activation, suggesting that miR-218-5p mediates the acquisition of osteomimicry properties of metastatic cells, and thus favouring their adaptation in bone [23]. Furthermore, exosomal miR-218 produced by MDA-MB-231 cells directly downregulates collagen type I alpha 1 (COL1A1) in MC3T3 pre-osteoblastic cells, which leads to a diminished type I collagen deposition by mature osteoblasts *in vitro* [24].

### miR-940

The oncomiR miR-940—secreted by cancer cells—induces osteoblastic bone metastasis [25]. MiR-940 is another good example of miRNAs originating from cancer cells that is responsible for modifications of bone remodelling. As aforementioned for miR-218 [23], miRNAs can be secreted from cells—commonly, via exosomes—and mediate the cross-talk between cancer cells and bone microenvironment [16]. MiR-940 is highly expressed in exosomes from prostate cancer cell lines that induce an osteoblastic lesion in comparison to cells lines that cause osteolytic lesions [25]. The exosomal cancer cell-derived miR-940 is able to promote the osteogenic differentiation of human mesenchymal stem cells from which osteoblast lineage originates [25]. Interestingly, when miR-940 is experimentally over-expressed in a breast cancer cell line, the MDA-MB-231, which usually induces osteolytic lesions in animals, these cells induce osteoblastic lesions in vivo [25]. This study clearly shows how a single miRNA, produced by cancer cells, once secreted within exosomes, can affect the activity/maturation of normal recipient cells in the bone microenvironment. These data suggest that not only miRNA dysregulations within cancer cells are important to study but also the alteration of circulating miRNA levels that can worsen pathological conditions, as shown in this study, or that could prepare a fertile soil for the seeding of disseminated cancer cells in distant organs.

## Oncosuppressor miRNAs in breast cancer bone metastasis

### miR-30 family (miR-30s)

The five members of the miR-30 family (miR-30s)—miR-30a, miR-30b, miR-30c, miR-30d and miR-30e—act as oncosuppressors in breast cancer [26]. MiR-30s low expression levels in primary breast tumours are associated with poor clinical outcomes [26]. Additionally, miR-30s expression levels have been reported to be lower in human bone-tropic MDA-B02 breast cancer cells, a sub-population of the parental MDA-MB-231 cells, and in the BC-M1 cells, a disseminated tumour cell line isolated from the bone marrow of a breast cancer patient, when compared to MDA-MB-231 cells [26]. Interestingly, the forced expression of miR-30s in MDA-B02 cells impedes both skeletal tumour progression and bone destruction in animal models [26]. MiR-30s are negative regulators of key oncogenes and pro-osteoclastic factors that contribute to bone metastasis development (IL-8, IL-11, DKK-1, RUNK2, CDH11, CTGF and integrins ITGA5 and ITGB3) [26]. *Cdh11* or *Itga5* silencing in bone-tropic breast cancer cells recapitulates the inhibitory effect of miR-30s on bone metastasis formation in animal models, further confirming that these effector oncogenes are regulated by miR-30s [26].

A recent study has reported that high circulating miR-30b-5p levels are detected in patients with metastatic breast cancer (*n*=12) compared to patients with localised breast cancer (*n*=20) [27]. Higher miR-30b-5p levels in metastasis specimens (*n*=22), including bone metastasis, were also observed when compared to matched primary tumours (*n*=16) [27]. The authors [27] hypothesised that, at an early stage of metastasis development, miR-30b-5p is downregulated in metastatic cancer cells undergoing epithelial-to-mesenchymal transition (EMT), whereas it is re-expressed in later stages of the metastatic process, when cancer cells undergo mesenchymal-to-epithelial transition (MET), suggesting a time-dependent role for miR-30b-5p in breast cancer. However, the limited number of patients evaluated in this study clearly prevents any firm conclusion. Furthermore, accumulating evidence demonstrates that miR-30 family members act as oncosuppressor genes in breast cancer progression and metastasis [26, 28, 29]. Nevertheless, this study [27] highlights the potential use of miRNAs as biomarkers—beyond their potential use as therapeutic agents or targets—taking in account that miRNAs are stable and can be easily detected in the circulation of cancer patients [16].

### miR-34a

MiR-34a attenuates tumour growth in TNBC, the most lethal subtype of breast cancer that often metastasises to distant organs including bone [30, 31]. MiR-34a expression is lost in TNBC cells, in particular in claudin-low TNBC breast cancer cell lines, which express mesenchymal and stem cell markers, as well as in primary TNBC tumour specimens [30]. Moreover, when compared to MDA-MB-231 cells, very low miR-34a-5p expression levels are observed in 1833 cells, a bone-metastatic sub-population of the MDA-MB-231 parental cell line [30]. The forced expression of miR-34a in MDA-MB-231 and 1833 breast cancer cells inhibits cell proliferation and invasion, activates cell senescence, and promotes sensitivity to dasatinib, a BCR-ABL and Src tyrosine kinase inhibitor [30, 32]. Systemic miR-34a administration reduces subcutaneous or orthotopic growth of induced tumours in animal models [30]. In human, a phase-I clinical trial has been conducted and patients with refractory advanced solid tumours (*n*=47) were treated with escalating doses of a miR-34a mimic encapsulated in lipid nanoparticles, called MRX34 [33]. Although this clinical trial was terminated prematurely due to serious adverse events in patients, including infusion-associated events that could be linked to the liposome carrier, it showed preliminary evidence of an anti-tumour activity [29]. A better strategy of delivery should be investigated together with the possibility of a combined therapy with other miRNAs or approved drugs to increase the efficiency of treatments for the still incurable TNBC.

### miR-124

MiR-124 has been largely investigated for its role as a tumour suppressor in different types of cancer and, more recently, as a critical factor in breast cancer metastasis in bone [34]. MiR-124 expression is decreased in human breast cancer cell lines in comparison to normal human mammary tissue, and further decreased in invasive breast cancer cell lines, including bone-seeking cancer cells, compared to their non-invasive counterpart [34]. The clinical relevance of miR-124 is further confirmed by the fact that miR-124 expression—evaluated by in situ hybridisation—is reduced in primary breast tumours in comparison to paired non-tumour tissues, and even more reduced in metastatic bone tissues [34]. The forced expression of miR-124 in bone-tropic breast cancer cells reduces bone metastasis formation and the extent of osteolysis in vivo, whereas miR-124 inhibition in non-invasive cancer cells promotes bone metastasis formation [34]. Mechanistically, the downregulation of miR-124 expression levels result in increased levels of its direct target IL-11, which acts as an effector to promote osteoclastogenesis and bone metastasis formation [34].

### miR-125b

MiR-125b regulates key players of the bone microenvironment with a role in breast cancer bone metastasis formation [35]. In bone, the hypoxia inducible factor-1 alpha (HIF1A) has a promoting effect on breast cancer metastasis by upregulating prostaglandin endoperoxide synthase-2 (PTGS2, also known as COX-2) [35]. The forced expression of miR-125b in bone-tropic human 1833 breast cancer cells reduces bone metastasis development by downregulating ETS1—a player for the invasive program of cancer cells—and the extent of osteolytic lesions in vivo [35]. Interestingly, a stronger inhibitory effect is observed when the forced expression of miR-125b is combined with the treatment of animals with the chemotherapeutic agent NS-398, an inhibitor of the pro-tumorigenic factor PTGS2 [35]. In vitro, miR-125b expression increases the expression of E-cadherin, an epithelial cellular marker, and it prevents MMP2 and vimentin expression, two mesenchymal cellular markers [35]. The authors [35] suggest that miR-125b might affect the interaction between ETS1 and HIF1A, thus impairing (directly and/or indirectly) PTGS2. A combined therapeutic approach NS-398/miR-125b needs further validations but seems promising.

### miR-135 and miR-203

MiR-135 and miR-203 are two oncosuppressors of breast cancer and metastatic bone disease [36]. In breast, miR-135 and miR-203 are highly expressed in normal mammary epithelial cells, while they are poorly expressed in bone metastasis specimens of breast cancer patients in comparison to healthy bone [36]. Moreover, miR-135 and miR-203 are highly expressed in non-metastatic MCF-7 breast cancer cells and poorly expressed in metastatic MDA-MB-231 breast cancer cells [36]. The forced expression of miR-135 and miR-203 in MDA-MB-231 cells suppresses their migratory properties in vitro by downregulating genes involved in cell motility (*Rock*, *Cd44* and *Ptk2*) [36]. In vivo, the ectopic expression of miR-135 and miR-203 in MDA-MB-231 cells reduces the size of primary tumours and the spontaneous formation of metastases, including bone metastases [36]. MiR-135 and miR-203 expression also protects against the formation of osteolytic lesions in metastatic animals by directly targeting RUNX2 in MDA-MB-231 cells, a transcription factor that promotes early stages of tumour progression and enhances the formation of osteolytic lesions in animals bearing bone metastases [36]. MiR-135 also directly targets *Smad5*, a component of the BMP signalling pathway, which consequently reduces the expression of a BMP-2 downstream gene, *Id2* [36]. Together, these findings pointing out two different molecular mechanisms that can explain, at least in part, the role of miR-135 and miR-203 as oncosuppressors in breast cancer bone metastasis.

### miR-205

MiR-205 mediates a molecular mechanism involved in breast cancer bone metastasis [37]. Transglutaminase-2 (TG2) induces EMT in various tumours, and its overexpression in human MCF-7 breast cancer cells (MCF7/TG2-C277S) promotes bone metastasis formation in *nude* mice [37]. Interestingly, TG2 directly targets miR-205 [37]. The forced expression of miR-205 in MCF7/TG2-C277S cells drastically inhibits bone metastasis formation in vivo, and suppresses the expression of ZEB1—an EMT-related factor—normally induced by TG2 in vitro [37]. Of note, miR-205 (like miR-30s) also targets the integrin ITGA5, which plays an essential role in mediating breast cancer bone metastasis formation [26, 38]. The inhibition of TG2 or ITGA5 and/or the expression rescue of miR-205 could therefore be potential therapeutic approaches for the treatment of metastatic breast cancer patients with a TG2 dysregulation [37].

### miR-429

MiR-429 is a member of the miR-200 family, which members are known for their oncosuppressor roles in cancer progression [39]. Expression levels of miR-429 are low in bone tissues from metastatic breast cancer patients in comparison to primary tumour tissues, and miR-429 low expression positively correlates with poor bone metastasis-free survival of breast cancer patients [40]. The conditioned medium from a bone metastatic sub-population of MDA-MB-231 cells, the 231-B cell line, that artificially over-expresses miR-429, promotes OPG and reduces RANKL expression levels in MC3T3-E1 osteoblastic cells in comparison to control [40], suggesting that this miRNA promotes osteogenic differentiation in vitro. MiR-429 also acts on osteoclasts by directly targeting CrkL (v-CRK avian sarcoma virus CT10-homolog-like) and decreasing MMP-9 expression, which results in the inhibition of osteoclast differentiation in vitro [40]. In vivo, animals bearing 231-B breast cancer cells overexpressing miR-429 have less bone metastatic lesions than animals bearing parental cancer cells [40]. It is suggested that miR-429 and CrkL could be used as biomarkers for predicting bone metastasis in breast cancer patients at an early stage.

## Concluding Remarks

MicroRNAs (miRNAs) are key regulators of the formation and progression of breast cancer bone metastasis. MiRNAs act at different levels within metastatic breast cancer cell—in situ regulation—or affecting the differentiation and activity of bone resident cells when they physically interact with them or at long distance. In fact, the expression of specific miRNAs can be altered in bone-metastatic breast cancer cells and enhance cell migratory and proliferative properties as well as drug-resistance or stem-like phenotypes to promote the seeding and adaptation of cancer cells in bone. Additionally, breast cancer-derived circulating miRNAs can act at long distance to educate and remodel the bone microenvironment making it more permissive for the seeding of disseminated cancer cells, and disseminated cancer cells in bone can then physically interact with bone cells and alter their physiological activities in order to further sustain cancer cell growth.

Since the role of miRNAs in bone metastasis is very central, it has been proposed to rescue their expression levels when the miRNA is downregulated or inhibit their expression levels once upregulated in metastatic breast cancer cells, or to modulate expression levels of circulating miRNAs. Experimentally, it has been proven that, by manipulating even a single miRNA, it is possible to modify the phenotype of cancer cells or modify their effects on the bone microenvironment. However, while these experimental procedures are very effective, we are still far to have an efficient delivery method to treat cancer patients with a ‘miRNA therapy’. The phase-I trial with MRX34 was a partial success, and new delivery methods must be explored. Furthermore, since miRNAs are stable in biological fluids and easily detected, they could be used as diagnostic/prognostic tools in the clinic to predict early relapse or response to specific treatments. Hopefully, in the forthcoming years, the exciting field of miRNA research will progress, and miRNAs will be part of an armamentarium of advanced and personalised therapies for breast cancer patients at high risk of relapse in bone.

## Summary

The over-expression of specific miRNAs (*e.g.* miR-10b, miR-21 and miR-218-5p) or their downregulation (*e.g* miR-30s, miR-34a, miR-124, miR-125b, miR-135, miR-203, miR-205 and miR-429) in breast cancer cells promotes the formation of metastasis in bone [17, 19, 23, 26, 30, 34–37, 40].

MiRNAs released by bone cells (*e.g.* the osteoclast-derived miR-214-3p) or breast cancer cells (*e.g.* miR-940) can affect bone metastasis formation [16, 25].

A ‘miRNA therapy’ with a miRNA mimic (MRX34) has been evaluated in a phase-I trial, but adverse effects have been reported, likely due to the liposome carrier [33]. Better strategies of miRNA delivery should be explored.

MiRNAs can be detected in the biofluids (*e.g.* serum) of breast cancer patients and can be used as diagnostic biomarkers [27].

### Availability of data and material

Not applicable.

### Code availability

Not applicable.
